# Efficacy of non-pharmacological treatments in pediatric MASLD: a review of the literature

**DOI:** 10.3389/fnut.2025.1667377

**Published:** 2025-11-12

**Authors:** Antonella Mosca, Maria Rita Braghini, Andrea Pietrobattista, Anna Alisi, Andrea Vania

**Affiliations:** 1 Unit of Hepatology and Transplant Clinic, Bambino Gesù Children’s Hospital, IRCCS, Rome, Italy; 2 Research Unit of Genetics of Complex Phenotypes, Bambino Gesù Children’s Hospital, IRCCS, Rome, Italy; 3 Independent Researcher, Rome, Italy

**Keywords:** MASLD, pediatric, non-pharmacologic therapy, physical activity, Mediterranean diet

## Abstract

Metabolic dysfunction-associated steatotic liver disease (MASLD), previously known as non-alcoholic fatty liver disease (NAFLD), comprises a spectrum of liver disorders characterized by excessive accumulation of triglycerides in hepatocytes. There are multiple treatment strategies proposed for pediatric MASLD, with lifestyle modifications and a balanced dietary regimen – particularly the Mediterranean diet in combination with weight loss, when needed – currently representing the cornerstone of clinical management. Behavioral psychological interventions are gaining increasing importance particularly in addressing comorbid eating disorders and supporting long-term adherence to therapeutic regimens. In addition, in daily clinical practice, the use of food supplements with antioxidant and hepatocyte anti-inflammatory effects is indicated in more advanced stages of steatosis, with the aim of limiting disease progression toward high-grade fibrosis and/or cirrhosis. This review aims to summarize current behavioral and nutritional therapeutic approaches for the management of pediatric MASLD.

## 1 Introduction

Metabolic dysfunction-associated steatotic liver disease (MASLD), also sometimes referred to as metabolic dysfunction-associated fatty liver disease (MAFLD), and previously known as nonalcoholic fatty liver disease (NAFLD), encompasses a continuum of hepatic disorders characterized by excessive lipid accumulation in hepatocytes (1). In light of the recent change in nomenclature from NAFLD to MASLD/MAFLD, this review will adopt the historical term NAFLD whenever referring to studies published prior to the redefinition. The clinical spectrum of MASLD ranges from simple steatosis to more advanced and severe stages, including metabolic dysfunction-associated steatohepatitis (MASH), fibrosis and, ultimately, cirrhosis, each stage carrying a progressively greater risk of hepatic impairment (2). Addressing liver steatosis and fibrosis is critical to arrest disease progression and avert life-threatening sequelae such as cirrhosis, hepatic failure, and hepatocellular carcinoma. Moreover, appropriate management may help mitigate extra-hepatic complications, including cardiovascular morbidity (3).

Treatment strategies for MASLD are inherently multifaceted, and lifestyle changes, particularly a Mediterranean-like diet and weight loss, represent the mainstay of management (4). In pediatric populations, pharmacological interventions remain limited, and clinical trials are relatively scarce. In adults, pharmacotherapy targets both metabolic dysregulation and hepatic pathophysiology, with agents such as Glucagon-like peptide-1 (GLP-1) receptor agonists, peroxisome proliferator-activated receptor (PPAR) agonists, fibroblast growth factor 21 (FGF21) analogs, and selective thyroid hormone β receptor agonists (e.g., resmetirom). Recently, resmetirom was the first medication approved by the Federal Drug Administration (FDA) specifically for the treatment of adults with non-cirrhotic MASH with moderate to advanced liver fibrosis (5); while several drugs are currently underway in phase III trials for adults with MASH after have demonstrated beneficial effects on both resolution of MASH and improvement of fibrosis, including the GLP-1 receptor agonists semaglutide and survodutide, the PPAR agonist lanifibranor and the FGF21 analog efruxifermin (6–9). However, the heterogeneity of MASLD poses significant challenges in defining universally effective treatments for children. Despite promising clinical trial data, the therapeutic management of pediatric MASLD remains a scientific challenge, with no approved pharmacological treatments currently available for pediatric population, where the identification of MASH condition and/or fibrosis as present earlier makes the disease more aggressive than in adults. Pediatric MASLD also exhibits unique features compared to its adult counterpart with regard to prevalence, histological characteristics (e.g., a different pattern of liver inflammation), diagnosis and treatment approaches. Furthermore, evidence suggests that pediatric MASLD has a strong hereditary component, with genetic variations interacting closely with environmental factors to influence disease progression. Overall, these features make lifestyle changes the primary therapeutic intervention for these children. The purpose of our review is to consolidate and critically examine the spectrum of non-pharmacological therapeutic options for pediatric MASLD.

## 2 Research materials and methods

A comprehensive literature search was conducted from inception to June 2025 across the following electronic databases: PubMed, Embase, Cochrane Central Register of Controlled Trials (CENTRAL), and Web of Science. The research strategy was developed using a combination of Medical Subject Headings (MeSH) and free-text keywords pertaining to MASLD and its synonyms and relevant therapeutic interventions. The detailed search strategy for PubMed/MEDLINE is outlined below, with similar strategies adapted for other databases:

((“Metabolic Dysfunction-Associated Steatotic Liver Disease”[Mesh] OR “Non-alcoholic Fatty Liver Disease”[Mesh] OR “MASLD” OR “NAFLD” OR “MASH” OR “NASH” OR “fatty liver”) AND (“Child”[Mesh] OR “Adolescent”[Mesh] OR “pediatric*” OR “pediatric*” OR “children” OR “adolescent*”) AND (“Lifestyle*” OR “Diet Therapy”[Mesh] OR “Exercise”[Mesh] OR “Mediterranean Diet” OR “physical activity” OR “behavioral therapy” OR “psychological support” OR “omega-3” OR “vitamin D” OR “probiotics” OR “nutraceutical*”)). Inclusion Criteria: Studies conducted in patients aged 2 to 18 years with a diagnosis of MASLD/NAFLD confirmed by imaging (ultrasound, MRI-PDFF), elastography, or histology (liver biopsy). Studies evaluating any non-pharmacological intervention, including but not limited to: Dietary interventions (e.g., Mediterranean diet, low fat/sugar diet). Structured or unstructured physical activity programs. Behavioral or psychological interventions (e.g., cognitive-behavioral therapy, family approach). Supplementation with nutraceuticals (e.g., omega-3 fatty acids, vitamin D/E, probiotics). Also, the studies with a control group (i.e., placebo, standard care, no intervention) or pre-post studies without a control group. Randomized controlled clinical trials (RCTs), non-randomized clinical trials, prospective observational studies (cohort), and pre-post studies.

The Exclusion Criteria were the studies on secondary causes of fatty liver disease (e.g., alcohol, drugs, viral hepatitis, genetic disorders). Editorials, letters, case reports, commentaries and reviews of the literature are not systematic. Studies that evaluate exclusively pharmacological interventions. Duplicate data or secondary publications of the same study, abstracts at conferences (only the most comprehensive publication was included).

**Figure 1:**
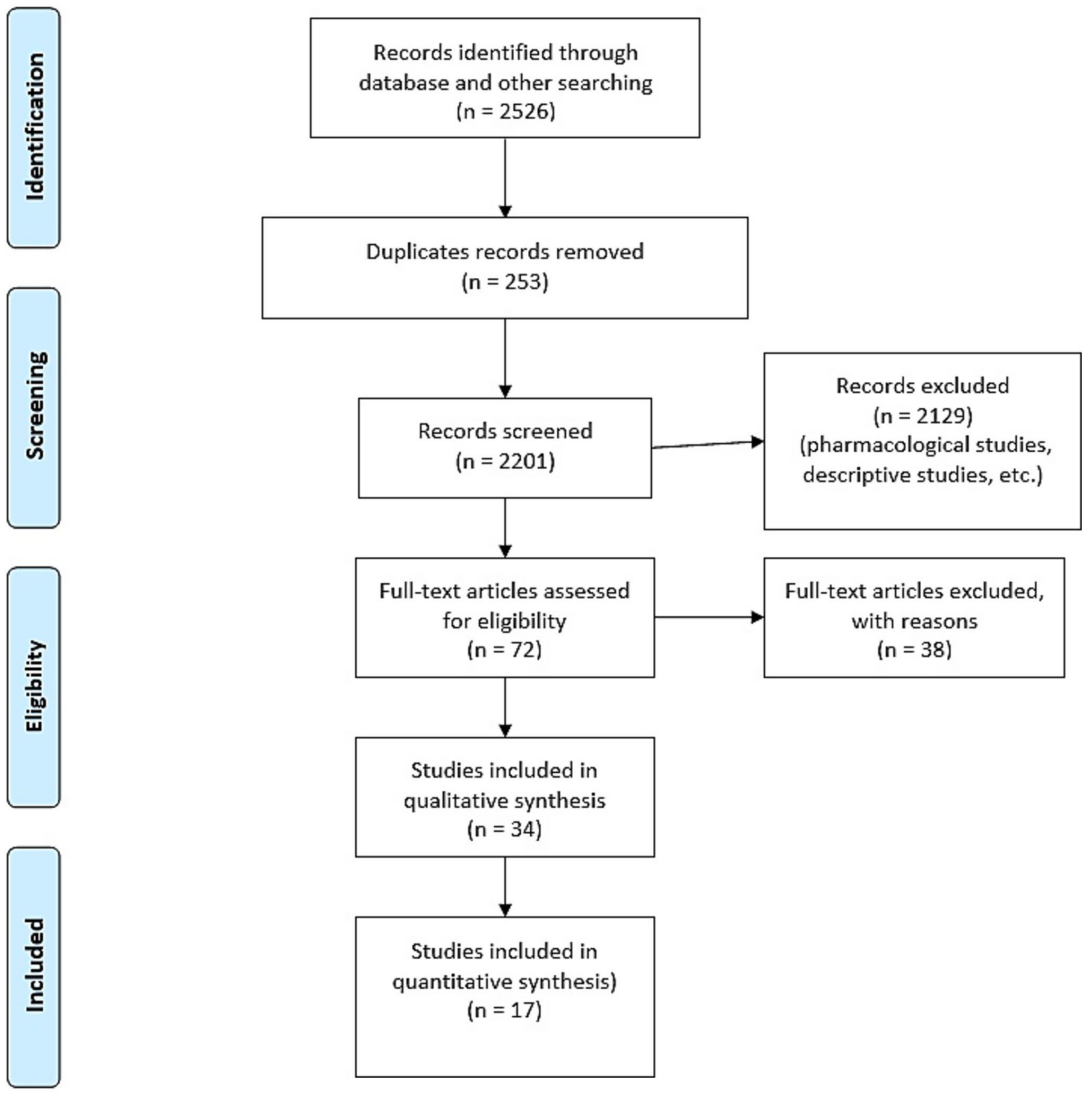
Flowchart PRISMA 2020 checklist.

This review was designed and reported in accordance with the PRISMA 2020 checklist (Flowchart, Figure 1).

## 3 The heart of therapy: lifestyle modifications

The non-pharmacological strategies are primarily centered on lifestyle interventions, targeting the correction of metabolic risk factors and the promotion of hepatic health.

A balanced and healthy diet combined with regular physical activity remains the primary strategy for managing both pediatric and adult patients with MASLD (5, 10). In this context, it has been demonstrated that regular physical activity alongside a diet closely following the principles of the Mediterranean diet improves ALT levels and the ultrasound appearance of steatosis. Indeed, weight reduction has been associated with significant changes in serum ALT levels and improvement in liver steatosis both in children and adults with MASH (11, 12). However, individual responses to and adherence with lifestyle interventions vary widely, making it difficult to define an optimal dietary or exercise program for everyone. Therefore, a personalized approach and family-based lifestyle education are crucial. In the Western world, current dietary habits and lifestyle factors necessitate culturally adapted lifestyle interventions. Family-based programs should prioritize reducing sugar-sweetened beverage consumption, increasing dietary fiber intake and promoting physical activity to ensure long-term adherence and effectiveness (13).

Here, we summarize the key findings regarding lifestyle interventions for pediatric patients with MASLD. These interventions include dietary changes (primarily based on the Mediterranean diet), physical activity, and holistic, family-centered approaches. The completed randomized controlled trials (RCTs) conducted on pediatric patients with MASLD are discussed and listed in Table 1.

**Table 1:** Summary of RCTs on lifestyle interventions for pediatric MASLD (2010–2025).

Intervention (Type)	ClinicalTrials.gov ID	Intervention group	Control group	Study population	Follow up duration	Key findings	Notes/limitations	Institute
Dietary modification	NCT05499585	Whole dairy	Habitual diet	20 children with NAFLD aged 10–17	24 weeks	Results not found	Lack of published results	University of California, San Diego
Dietary modification	NCT02513121 (24)	Low sugar diet	Habitual diet	40 children with NAFLD aged 11–16	8 weeks	Improvement in hepatic steatosis, fasting insulin, and ALT levels	Short follow-up durations	University of California, San Diego
Dietary modification	NCT05073588 (25)	Indo-Mediterranean diet	Calorie restricted diet	39 children with NAFLD aged 8–18	180 days	Indo-Mediterranean diet was superior to calorie restricted diet to reduce CAP values and BMI	Small sample size	Institute of Liver and Biliary Sciences, India
Dietary modification	NCT04415112 (26)	Mediterranean diet	Low fat diet	45 children with NAFLD aged 9–17	12 weeks	Both diets improved steatosis to a similar degree	Small sample size	Antalya Training and Research Hospital
Dietary modification	NCT04561804 (27)	Low carbohydrate, low glycemic load, and isocaloric diet	Conventional treatment	100 children with NAFLD aged 7–18 ongoing weight loss reduction surgery	6 months	Improvement in hepatic fibrosis	Surgery outcome	Tel-Aviv Sourasky Medical Center
Dietary modification	NCT06579729	Low free sugar diet	Habitual diet	146 children with NAFLD aged 5–14	48 weeks	Results not found	Lack of published results	China Medical University, China
Dietary modification	NCT00585299	Low fat diet	Habitual diet	4 children with NAFLD aged 10–21	16 weeks	Results not found	Lack of published results and several small sample size	Yale University
Dietary modification	NCT00480922 (28)	Low glycemic load diet	Low fat diet	40 children with NAFLD aged 8–17	6 months	Both diets improved steatosis to a similar degree	Small sample size	Boston Children’s Hospital
Exercise intervention	NCT02258126 (29)	Exercise (3 days/week) and healthy lifestyle education program	Healthy lifestyle education program	115 children with NAFLD aged 9–11	6 months	Exercise training in multicomponent intervention programs contributed to improvement of steatosis, adiposity and insulin resistance	The study design was not randomized	University of the Basque Country (UPV/EHU)
Lifestyle intervention	NCT02412540	Dietary, behavioral and activity interventions designed to reduce weight	Weight loss surgery	62 children with NAFLD aged 12–19	12 months	Results not found	Lack of published results	Children’s Hospital Medical Center, Cincinnati

RCT, randomized controlled trial; MASLD, Metabolic Dysfunction-Associated Steatotic Liver Disease; NAFLD, non-alcoholic fatty liver disease; ALT, alanine aminotransferase; CAP, controlled attenuation parameter; BMI, body mass index.

### 3.1 The Mediterranean diet

The Mediterranean diet (MedDiet), characterized by a low intake of simple sugars, a balanced lipid profile and high levels of antioxidants and dietary fiber, represents an optimal nutritional strategy for the management of MASLD. This dietary pattern has been extensively studied, and its beneficial effects are well documented and widely acknowledged in the scientific literature (12, 14).

The beneficial effects of the MedDiet on fatty liver disease are attributed to compounds such as polyphenols, vitamins, dietary fiber, and other molecules with anti-inflammatory and antioxidant properties. These have been associated with reductions in insulin resistance, inflammation, and oxidative stress in MASLD (15).

It is estimated that about 60% of hepatic triglycerides (TGs) originate from free fatty acids released by adipose tissue, 26% result from *de novo* lipogenesis, and 15% are directly derived from dietary intake (16, 17). The MedDiet’s fatty acid profile, rich in mono- and polyunsaturated fatty acids (PUFAs) and low in saturated fats, appears to attenuate liver steatosis by improving serum lipid levels, enhancing lipid oxidation, modulating hepatic gene expression, and inhibiting *de novo* lipogenesis (1, 16). Dietary intake of PUFAs, especially omega-3 s, plays a crucial role in reducing hepatic lipid accumulation. Olive oil, a central component of the MedDiet, contains hydroxytyrosol, a polyphenol with documented efficacy in attenuating oxidative stress and liver inflammation. Likewise, high consumption of fruits and vegetables – another hallmark of the MedDiet – is frequently associated with reductions in liver inflammation and lipid deposition. Collectively, these bioactive constituents not only contribute to the reduction of liver fat but also aid to normalize liver enzymes and thus to improve liver function (18).

Several interventional RCTs and observational studies have been conducted to investigate the efficacy of the MedDiet alone on metabolic parameters of pediatric and adult patients with MASLD.

Two distinct meta-analyses, which examined the effect of the MedDiet on the liver enzymes of adult patients with MASLD, highlighted how adherence to the MedDiet led to greater improvement in anthropometric status, lipid profile, insulinemic and glycemic profile, and hepatic indices. Adherence to this dietary pattern was also associated to significant improvements in key hepatic biomarkers related to MASLD, including ALT, aspartate aminotransferase (AST), and gamma-glutamyl transferase (GGT) (19, 20). The RCTs conducted in adults have typically lasted from 6 to 24 weeks and have consistently demonstrated that adherence to the MedDiet is associated with significant reductions in intrahepatic fat content, improved insulin sensitivity, and amelioration of cardiovascular risk factors, when compared to control groups and low-fat dietary regimens (19).

Also for the pediatric population, several RCTs have shown a significant reduction in both transaminase levels and insulin resistance (21, 22). A recent meta-analysis showed that adherence to MedDiet significantly improves liver function by reducing serum transaminase levels in children with MASLD. Nevertheless, the authors underscored the low quality of some included studies and emphasized the need for further high quality RCTs (21). In a separate literature review, Trobia et al. also identified the MedDiet as one of the most thoroughly studied and widely recommended dietary interventions for managing pediatric MASLD. The benefits of this diet were attributed to its richness in unsaturated fats – such as those found in olive oil – fiber, antioxidants and a balanced ratio of omega-3 to omega-6 fatty acids. The authors also stressed the pivotal role of weight loss, which is often more easily achieved through adherence to the MedDiet (23). Overall, while implementation of MedDiet-based interventions outside the Mediterranean region remains inconsistent, available evidence suggests hepatic and metabolic improvements associated with MedDiet adherence even in the absence of significant weight loss or when weight loss is similar between intervention and control groups (20, 22).

Other dietary interventions have been explored in the context of RCTs for pediatric MASLD, as listed in Table 1. These interventions mainly examined the effects of low-sugar, low-carbohydrate, low-glycemic load and low-fat diets. The results suggest that these dietary modifications reduce hepatic fat and improve metabolic parameters in children with MASLD (24–29). However, the current landscape of randomized and interventional trials in children with MASLD remains limited, most studies have a small sample size, analyzed outcomes are heterogeneous, and follow-up durations are short.

Alternative dietary approaches, including the ketogenic diet, intermittent fasting and soy-based diets, have been proposed in smaller-scale studies, but their large-scale applicability remains to be established, particularly within pediatric populations.

Overall, these findings highlight the need for larger, multicentre RCTs with standardized endpoints to determine the long-term effectiveness and safety of different dietary interventions.

### 3.2 Physical activity

A sedentary lifestyle has long been associated with the development of MASLD. Children and adults individuals affected by MASLD tend to exhibit greater sedentary behavior and significantly lower levels of physical activity, day by day, than healthy controls, thereby reducing their active energy expenditure by as much as 40% (23, 30). Notably, in individuals aged 20 years and older each additional hour/day of sedentary behavior has been associated to a 4% higher risk of MASLD (31). Conversely, higher levels of physical activity – over 500 metabolic equivalents (METs)-minutes/week – have been associated with a reduction of up to 57% in MASLD risk, along with lower intrahepatic lipid content and improved metabolic profile, irrespective of weight loss (23, 30, 31). Current clinical practice guidelines for the management of MASLD advocate for weight loss achieved through calorie-restricted diets and physical activity. In this regard, the synergistic effect of moderate calorie restriction (−500 kcal per day) and 30–60 min of moderate-intensity exercise performed 3–5 days per week have been shown to confer greater benefits on MASLD- related outcomes than intervention alone (32, 33). A single-arm interventional study evaluated the impact of a multifaceted, structured lifestyle program on 403 U. S. adults (mean age 54 years) with Metabolic Syndrome (MetS) and MASLD, as part of the University of Michigan’s Metabolic Fitness (MetFit) initiative (21). This 24-week intervention included 45 min of aerobic exercise once a week complemented by 45 min per week of nutritional education focused primarily on MedDiet. The program aimed to achieve 5 and 10% weight loss after 12 and 24 weeks, respectively. Participants with MASLD showed significantly greater reductions in TGs (mean change: −45 mg/dL vs. −23 mg/dL) and ALT serum levels (−11 U/L vs. −3 U/L) after 24 weeks. Improvements in central adiposity and fasting glycaemia, and greater reduction in insulin resistance, TGs, and ALT levels were associated with ≥5% weight loss (34).

In the pediatric context, a meta-analysis by Giannini et al. (35) demonstrated that supervised exercise significantly reduced liver fat content compared to control groups. Lifestyle interventions that included structured and supervised exercise were also significantly associated with a marked reduction in the prevalence of NAFLD. Both vigorous and moderate-to-vigorous intensity both aerobic and resistance exercise, with a volume of ≥60 min/session and a frequency of ≥3 sessions/week, have been shown to confer benefits in terms of reduction of liver fat (35). These findings were further supported by several authors, which assessed the impact of exercise interventions on validated liver outcomes in youths aged 0–19 years diagnosed with NAFLD or at elevated risk. Five of the 11 studies evaluating fatty liver disease reported an absolute reduction of 1 to 3%. However, across the nine studies evaluating liver enzyme levels (transaminases), no statistically significant changes were consistently observed (36). The authors concluded that, although exercise shows potential, the current evidence base for its effectiveness in the treatment of pediatric MASLD remains limited due to methodological constraints, underscoring the need for further high-quality trials.

Exercise regimens should be individualized to align with each patient’s physical capacities and personal preferences. Regular moderate-intensity physical activity for 150–200 min per week, ideally distributed over 3 to 5 sessions, or an increase in activity level of over 60 min per week, can prevent or ameliorate MASLD. While exercise confers benefits in both overweight and lean individuals, it holds clinical relevance in cases of MASLD in lean subjects (37, 29).

### 3.3 Mental health and holistic and family centered approach

About 81% of patients with MASLD are present with obesity, while the remaining 19% are classified as having a “lean MASLD,” defined by a BMI < 75th percentile. The association between eating disorders and MASLD is particularly significant, with binge eating disorder being the most frequently observed, followed by anorexia and bulimia (38). Behavioral interventions targeting eating disorders have the potential to prevent progression to obesity and mitigate the risk of associated metabolic disorders, including type 2 diabetes, hypertension, and dyslipidemia. Behavioral interventions tailored to treat binge eating disorders not only address an underlying etiological factor but also contribute to sustained weight loss and reversal of MASLD and its associated complications. Moreover, such interventions enhance adherence to physical activity regimens, which remain a cornerstone of MASLD treatment (39). For these reasons, the integration of behavioral interventions into MASLD management is essential to address the complex interplay between psychological dysfunctions and obesity. This approach requires a careful evaluation of the patient’s dietary history and careful screening for the presence of eating disorders. Despite the clinical relevance of this association, the available data on the link between MASLD and eating disorders remains limited.

Eating disorders are multifactorial and multifaceted conditions influenced by a complex interplay of biological, psychological, environmental, and social determinants. The most widely used psychological intervention for binge eating disorder, endorsed by international clinical guidelines, is Cognitive Behavioral Psychotherapy (CBT). CBT is recognized as one of the most effective therapeutic approaches for eating disorders and is often considered a “transdiagnostic treatment” thanks to its applicability across a broad spectrum of disordered eating presentations, and its suitability for use with young patients (40). The theoretical foundation of CBT posits a shared maintaining mechanism among eating disorders: a dysfunctional self-evaluation that is disproportionately shaped by concerns over shape, weight and perceived control thereof. CBT focuses primarily on establishing regular eating patterns and actively engages patients in changing and stabilizing their eating pattern, while identifying triggers and early warning signs of relapse. Treatment can be delivered individually, in group settings, or through a blended format (39, 40). Beyond restructuring eating habits, behavioral therapy also equips patients with tools such as mindfulness, discomfort tolerance, interpersonal effectiveness, and emotion regulation to better manage their disorder.

The active involvement of the patient’s family and social network is crucial and should be recognized as a fundamental resource in the recovery process. Integrating these support systems enhances treatment adherence and long-term outcomes. In addition, a thorough understanding of the social dynamics that contribute to the development and maintenance of eating disorders is critical to dismantling the mechanisms that perpetuate the disease (41).

Targeted interventions aimed at enhancing self-esteem are crucial in pediatric patients, with the goal of improving the child’s self-perception by fostering appreciation for their personal strengths and achievements unrelated to their body weight. Furthermore, it is important to encourage the development of social skills, equipping children to manage challenging social situations, such as bullying, and to cultivate positive peer relationships. Motivational techniques, including realistic goal setting and recognition of progresses, play a key role in this process (40–42). The presence of a supportive and engaged social network is associated with sustained recovery and long-term maintenance of therapeutic gains.

### 3.4 Genetic implication

In family management, it is necessary to understand the role of genetics and epigenetics in the development of MASLD. In both children and parents, several genetic polymorphisms have been identified by several studies with important cofactors in the onset of MASLD and its progression. These genetic polymorphisms are genes implicated in the regulation of lipid accumulation in hepatocytes, oxidative stress, insulin resistance and fibrogenesis A high-frequency gene variant in MASLD is the PNPLA3 (Patatin-like phospholipase domain-containing protein 3) allele rs738409[G] (43). In addition to being the strongest determinant for the presence of MASLD compared to healthy controls, PNPLA3 rs738409[G] also conferred the highest risk of steatosis severity and modified the presence of portal fibrosis. The homozygous PNPLA3 rs738409[G] has been associated with a picture of zone 1-dominant steatosis and an increase in portal inflammation, as well as associated with a greater risk of evolution to fibrosis and cirrhosis, regardless of the presence of obesity (44).

Other known and possible SNPs to identify are: TM6SF2 (Transmembrane 6 superfamily member 2), the specific variant in this gene (rs58542926, E167K) is associated with greater fat accumulation in the liver and a higher risk of disease progression, its mechanism is linked to a reduced secretion of very low-density lipoproteins (VLDL) by the liver; MBOAT7 (Membrane Bound O-Acyltransferase Domain Containing 7), whose variant (rs641738) is a risk factor for the development and severity of MASLD, in particular for progression to MASH (45). KLB (Klotho-beta), which has been linked to increased inflammation and fibrosis in obese patients with fatty liver (46); SOD2 (Superoxide Dismutase 2), linked to the management of oxidative stress at the mitochondrial level, its variants can influence cell damage in the liver; and LPIN1 (Lipin 1) which plays a role in lipid metabolism and its variants have been associated with the disease (47). Importantly, having a genetic predisposition does not mean that you will develop the disease with certainty. However, knowledge of these genetic variants can help identify children at higher risk, who could benefit from closer monitoring and early, targeted interventions on diet and exercise.

## 4 Dietary supplements

**Figure 2:**
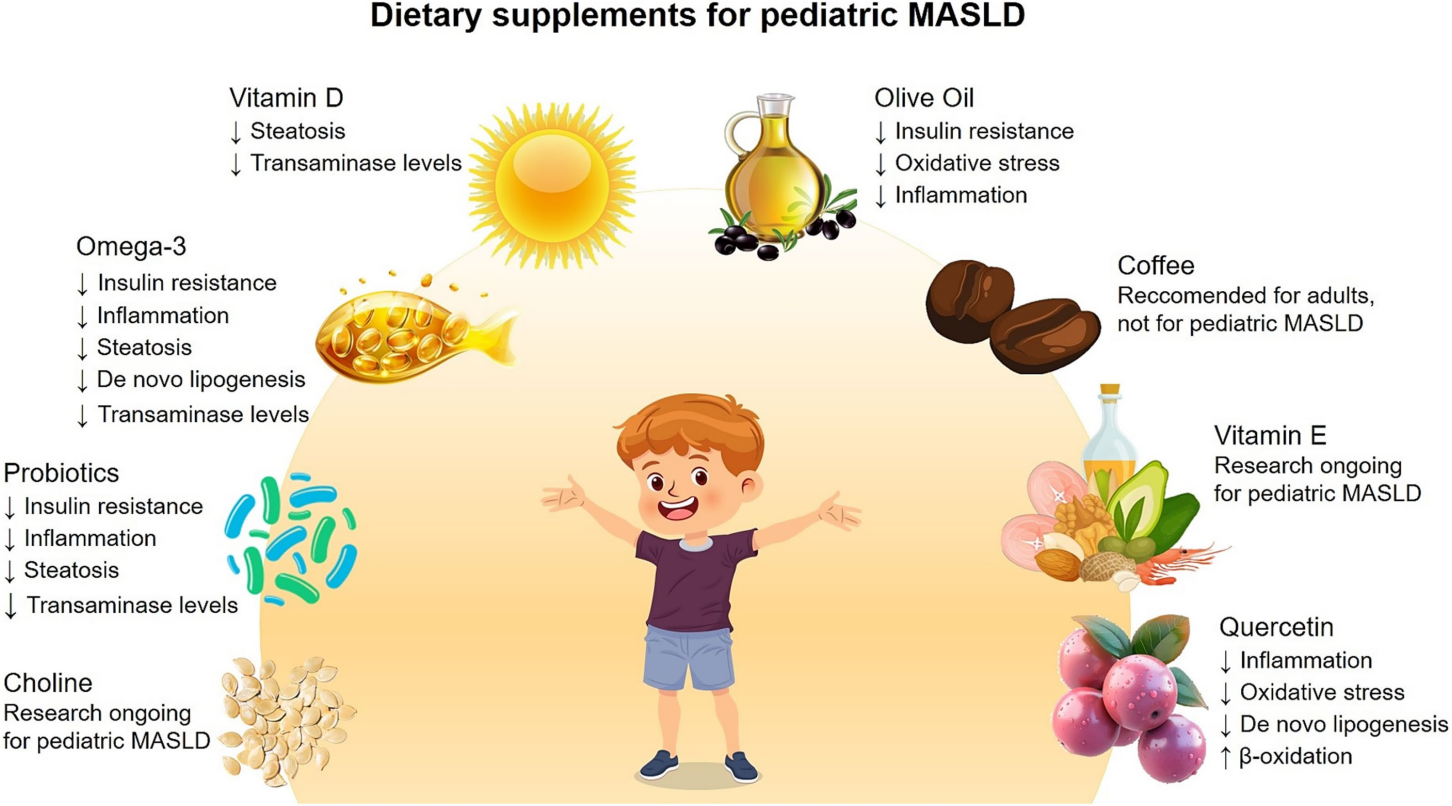
Dietary supplements for treatments of pediatric MASLD. Schematic representation of the main dietary supplements tested in pediatric patients with MASLD, and the key findings regarding their beneficial effects.

It is important to reiterate that, to date, no specific pharmacological agents have been specifically approved for the treatment of pediatric MASLD. When a pharmacotherapy is considered, it is typically reserved for selected cases with advanced liver fibrosis or other significant complications and is always used as an adjuvant to – rather than a substitute for – lifestyle modifications. Although research in this field is ongoing, lifestyle interventions remain the cornerstone of pediatric MASLD management. Recently, promising dietary supplements, including probiotics and long-chain omega-3 PUFAs, have been increasingly utilized in adolescents with NAFLD because of their antioxidant, anti-inflammatory, and metabolic properties (48). Here, we summarize the role of various nutraceuticals in pediatric MASLD treatment, as schematically represented in Figure 2.

### 4.1 Long-chain fatty acids-omega-3

Several evidences have demonstrated that long-chain omega-3 fatty acids, key regulators of liver gene transcription, can reduce fatty liver disease, enhance insulin sensitivity, and decrease markers of inflammation. Nobili et al. reported the results of a RCT based on the administration of docosahexaenoic acid (DHA), an omega-3 fatty acid of the PUFA family, in combination with diet and physical activity in 20 children with NAFLD. Six months of DHA supplementation resulted in improvements in BMI, insulin sensitivity index, serum ALT and triacylglycerol levels, and ultrasound-detected steatosis. Antisteatotic effects were also confirmed by liver biopsy at 16 months (49). DHA and docosapentaenoic acid (DPA) are two omega-3 PUFAs constituents of fish oil (FO). Supplementation with FO has been found to significantly improve liver function and lipid metabolism in patients with MASLD (50). The mechanism through which FO exerts therapeutic effects in MASLD are multifaceted. Firstly, FO enhances cellular membrane fluidity, which is positively correlated with the translocation of glucose transporter type 4 (GLUT4) into the cytoplasm. Increased membrane fluidity can simultaneously help restore the tyrosine kinase activity of insulin receptor substrates (IRS)-1 and −2, thereby facilitating insulin signal transduction (51). Another potential mechanism contributing to the development of MASLD is chronic low-grade inflammation, driven by the accumulation of hepatic TGs, which is associated with the recruitment of macrophages, leading to the synthesis of pro-inflammatory cytokines such as TNF-α, IL-1β and IL-6. Substantial evidence suggests that FO intervention inhibits the toll-like receptor (TLR)-4 signaling pathway, leading to a downregulation in the production of pro-inflammatory cytokines (5, 52). Furthermore, FO supplementation can improve MASLD by inhibiting TGs synthesis and promoting TGs oxidation. FO modulates various nuclear receptors (notably the PPAR family) and transcription factors (such as SREBPs) responsible for lipid synthesis and metabolism. PUFAs also regulate transcription factors that control the expression of enzymes involved in *de novo* lipogenesis, such as acetyl-CoA carboxylase (Acc) and fatty acid synthase (Fasn). This process inhibits de novo lipogenesis, which is a central contributor to fatty liver disease (52).

A recent meta-analysis indicated that subgroup analysis of ALT, AST, and GGT revealed markedly reduced heterogeneity when omega-3 dosage was kept below 2000 mg/day and treatment duration extended beyond 12 weeks, suggesting that heterogeneity is primarily attributable to these two parameters, and that those limits (i.e., no more than 2000 mg, and no less than 12 weeks) may represent the optimal FO treatment (53–55).

Available data indicate that the side effects of FO supplementation are minimal and comparable to placebo. Several studies have evaluated the effect of omega-3s in children with MASLD. A notable example is the RCT by Janczyk et al. (56), which demonstrated some positive effects on liver parameters in children with NAFLD following omega-3s supplementation. Other studies in children have shown improvements in TG levels and, in some cases, a modest reduction in liver fat and liver enzymes (ALT/AST), although results have not been consistently statistically significant across all histological endpoints (57–62). To date, omega-3s remain the most extensively studied lipid compounds with hepatic anti-inflammatory potential in hepatic disease.

### 4.2 Vitamin D

Vitamin D plays a crucial role not only in bone and calcium metabolism, but also in immune, anti-inflammatory and metabolic processes. Both in adults and children, a correlation was found between low vitamin D levels and the prevalence and severity of MASLD. Indeed, Vitamin D contributes to modulating MASLD through multiple pathways. The activation of vitamin D receptor, which is abundantly expressed in liver cells, has been shown to suppress hepatic lipid accumulation and inflammation. Vitamin D also influences insulin sensitivity, thus mitigating fat accumulation in the liver, and exhibits anti-inflammatory properties by inhibiting pro-inflammatory cytokines. Therefore, vitamin D deficiency may contribute to exacerbate MASLD pathological mechanisms (63).

Many observational studies have reported a higher prevalence of vitamin D deficiency in children with MASLD compared to healthy controls. While these findings support a potential association, they do not establish a direct causal relationship. Some interventional studies have examined the effect of vitamin D supplementation in children with MASLD and vitamin D deficiency, reporting improvements in serum vitamin D levels and, in some cases, a modest reduction in liver enzymes or steatosis (58, 64–66). However, there is still no strong and consistent evidence that vitamin D supplementation in children who are not deficient or only mildly deficient, in terms of significant improvement of MASLD or interruption of its progression. By contrast, when administered in combination with DHA, vitamin D has shown excellent results in attenuating the progression of liver damage in pediatric patients with MASLD (62).

### 4.3 Olive oil (and hydroxytyrosol)

Extra virgin olive oil, a cornerstone of the MedDiet, is widely recognized for its benefits on cardiovascular and metabolic health. It is particularly rich in monounsaturated fatty acids and phenolic compounds, such as hydroxytyrosol, which exhibits strong antioxidant and anti-inflammatory properties. It is hypothesized that regular consumption of olive oil may reduce insulin resistance, oxidative stress, and liver inflammation (64–67). Numerous studies, although not specific to olive oil as a single supplement, support the efficacy of the MedDiet in the management of pediatric MASLD, demonstrating improvements in liver enzymes, liver fat, and metabolic parameters (68).

Hydroxytyrosol, a potent polyphenol found in olive oil, has recently gained attention as a potential therapeutic agent in pediatric MASLD. Preliminary studies suggest that hydroxytyrosol supplementation may ameliorate fatty liver conditions in children with obesity (69, 70). However, research on the isolated use of olive oil or hydroxytyrosol as standalone interventions in pediatric MASLD is still in its early stages and requires evidence-based confirmation through well-designed, large-scale clinical trials to establish both its efficacy and safety. Nonetheless, current findings are encouraging.

### 4.4 Other dietary supplements

Many other supplements have been studied in the context of MASLD, with proposed mechanisms ranging from modulation of the gut microbiota to antioxidant action and the role of essential nutrients involved in lipid metabolism.

#### 4.4.1 Probiotics

The gut-liver axis plays a pivotal role in the pathogenesis of MASLD. Probiotics have been studied for their ability to modulate the gut microbiota and improve gut barrier integrity, thereby reducing systemic and hepatic inflammation (71).

The role of probiotics is to modulate the gut microbiota and improve its intestinal barrier function, thus reducing intestinal permeability and the subsequent translocation of bacterial toxins to the liver. Additionally, probiotics induce the production of short-chain fatty acids (SCFAs), notably butyrate, propionate, and acetate, which exert beneficial effects on liver metabolism and inflammatory responses, while also reducing insulin resistance (72).

Several RCTs and systematic reviews have explored the use of probiotics in pediatric MASLD, with mixed results. One of the earliest pediatric RCTs administered (LGG) to children with MASLD for 8 weeks, reported significant reductions in liver enzymes (ALT, AST), hepatic steatosis as assessed via ultrasound, and improvements in insulin sensitivity compared to the placebo group (73).

Sabico et al. (74) examined the effects of the administration of combined probiotics and prebiotics in adolescents with MASLD, reporting improvements in some metabolic markers and favorable shifts in the gut microbiota. Other RCTs, although generally showing positive trends, have failed to consistently achieve statistical significance across key parameters such as liver fat reduction, quantified by more precise methods (MRI, or histological improvements in liver biopsies) (75).

The wide heterogeneity in probiotic strains, dosages, treatment duration, and characteristics of the population studied (including age, MASLD severity, comorbidities) hinders direct comparison of the results across studies and limits the generalizability of findings. Some studies have shown no significant benefit over placebo.

#### 4.4.2 Vitamin E

Vitamin E is a potent antioxidant that can eliminate reactive oxygen and nitrogen species while increasing the activity of antioxidant enzymes. Thus, the potential of vitamin E as a dietary supplement has been extensively studied in MASLD, where oxidative stress plays an important role in the disease’s pathogenesis (76). In adults with MASH, the use of vitamin E has been studied and, in some cases, recommended (77). In children, several RCTs explored the possible benefits of vitamin E supplementation, however the evidence remains inconclusive, and its efficacy in pediatric MASLD is not universally accepted. While some studies suggest potential benefits, current pediatric guidelines generally refrain from endorsing its routine use, due to a lack of robust data and concerns about long-term safety (61, 78–80).

#### 4.4.3 Choline

Choline plays pivotal roles in various physiological processes and serves not only as a precursor to phospholipids and acetylcholine, but is also an essential nutrient involved in lipid metabolism. Its deficiency, which can be influenced by factors such as low intake, estrogen status and genetic polymorphisms, as well as its use as a substrate for trimethylamine production by certain intestinal bacteria, can contribute to the development of fatty liver disease. Although a few studies have explored choline supplementation in pediatric MASLD, its use as a dietary supplement is complicated by the fact that requirements are highly individualized and by the possibility that excessive phosphatidylcholine concentrations may increase the risk of cardiovascular disease in certain individuals. Therefore, although a RCT found benecial effects of its administration in combination with the omega-3 DHA and vitamin E in pediatric patients with NAFLD (61), the available evidence is limited and insufficient to support clinical recommendations, and biomarkers of choline status derived from metabolomics studies are required to clarify how choline supplementation can be incorporated into the diet to improve hepatic steatosis (81).

#### 4.4.4 Quercetin

This molecule is a natural flavonoid present in many plant foods (such as fruits, vegetables and tea), it is widely studied for its potential therapeutic properties, especially in the context of MASLD. Indeed, available studies suggest that quercetin can significantly improve MASLD through various mechanisms with reduction of lipid accumulation in the liver, as it appears to modulate hepatic lipid metabolism, reducing the synthesis of triglycerides and promoting their oxidation (82). It also has an antioxidant action, as it neutralizes free radicals and strengthens the body’s antioxidant defense systems, and anti-inflammatory action, decreasing the production of pro-inflammatory cytokines and the infiltration of immune cells in the liver. Finally, quercetin also appears to play a role in promoting mitophagy. One important mechanism is its ability to promote mitophagy (the process of selectively removing damaged mitochondria), often mediated by pathways such as AMPK, which is crucial for mitochondrial health and increases its antioxidant properties. All together, these properties make quercetin a promising supplement to be used in the prevention of liver dysfunction (83). A recent RCT found that the quercetin intervention decreased both intrahepatic lipid content and body mass index in adult patients with MASLD (84). However, the use of quercetin in pediatric MASLD is not currently recommended as a standard or first-line therapy. The lack of robust and specific clinical studies for children does not allow to establish its efficacy and safety in this age group.

#### 4.4.5 Coffee

The recent American Association for the Study of Liver Diseases (AASLD) guidelines suggest that the consumption of 3 or more cups of coffee per day could be recommended for patients with MASLD in the absence of contraindications (33). Indeed, coffee contains several bioactive compounds, including caffeine, chlorogenic acids, and diterpenes, which have been shown to have anti-inflammatory, antioxidant, and antifibrotic properties (85). Observational studies compared the risk of MASLD and MASLD-associated fibrosis in individuals who drank coffee or not, the results suggested that coffee consumption was associated with improvements in liver damage (86, 87). The beneficial effects seem to be independent of caffeine content (88) and a recent meta-analysis speculated that it may be due to an increase in adiponectin concentrations following coffee consumption (89). Despite these data, there are few RCTs investigating the effects of coffee consumption on the liver of adult patients with MASLD, and these tend to yield negative or inconclusive results. This makes it difficult to reach any definitive conclusions (90). Evidence in pediatric population are very limited. Although one RCT examined the impact of coffee or tea consumption on weight in adolescents (91), no RCT has specifically investigated the use of coffee or caffeine as a treatment for pediatric MASLD focusing on liver-related outcomes. Furthermore, pediatric organizations recommend that children under 12 should avoid caffeine and that adolescents should limit their caffeine intake due to their susceptibility to caffeine’s adverse effects (e.g., sleep disruption, anxiety, increased heart rate/blood pressure, gastrointestinal upset, possible behavioral effects, and the risk of dependence/withdrawal) (92). For these reasons, coffee or caffeine cannot be recommended as a treatment for MASLD in children at this time.

In summary, the role of dietary supplements in the management of pediatric MASLD shows an emerging but far from definitive picture. While certain supplements, such as omega-3 s fatty acids and, more recently, hydroxytyrosol from olive oil, have shown promise in preliminary studies, current evidence is often constrained by small sample sizes, heterogeneity of study protocols, and the paucity of large, long-term, and high-quality RCTs. Vitamin D appears to exert greater clinical benefit when combined with DHA for the direct treatment of MASLD in terms of attenuation of hepatic damage and disease progression. Table 2 summarizes a list of completed RCTs that used dietary supplements to treat pediatric MASLD.

**Table 2:** Summary of RCTs on nutritional supplementation for pediatric MASLD (2010–2025).

Supplement (Type)	ClinicalTrials.gov ID	Intervention group	Control group	Study population	Follow Up duration	Key findings	Notes/limitations	Institute
Omega-3 fatty acids (PUFAs)	NCT00885313 (59)	Omega-3 (DHA)	Placebo	60 children with NAFLD aged 4–16	24 months	Improved liver steatosis and insulin sensitivity	Small sample size	Bambino Gesù Hospital and Research Institute
Omega-3 fatty acids (PUFAs)	NCT02201160 (60)	Fish Oil Supplementation	Sunflower oil as a Placebo	30 children with NAFLD aged 8–18	6 months	Improved liver steatosis and related metabolic abnormalities	Short follow-up duration	St. Justine’s Hospital
Omega-3 fatty acids (PUFAs)	NCT01285362	Fish Oil Supplementation	Corn Oil as a Placebo	8 children with NAFLD under 18	12 months	Results not found	Small sample size and lack of published results	Columbia University
Omega-3 fatty acids (PUFAs)	NCT01547910 (56)	Fish Oil Supplementation	Sunflower oil as a Placebo	76 children with NAFLD aged 6–19	6 months	Improved AST and GGT levels	Short follow-up duration	Piotr Socha Children’s Memorial Health Institute
Vitamin E	NCT00655018 (78)	Vitamin E (alpha-tocopherol plus ascorbic acid)	Placebo	90 children with NAFLD aged 6–19	24 months	Did not seem to increase the efficacy of lifestyle intervention alone	Mixed age with young adult	Bambino Gesù Hospital and Research Institute
Vitamin E	NCT05905185 (79)	Vitamin E (Tocotrienol-rich fraction)	Placebo	29 children with NAFLD aged 10 to 18	6 months	Improved APO-A1, AST, and pro-inflammatory cytokine levels	Short follow-up duration	National University of Malaysia
Vitamin E	NCT00063635 (80)	Vitamin E or Metformin	Placebo	173 children with NAFLD aged 8–17	96 weeks	Neither vitamin E nor metformin was superior to placebo in attaining the primary outcome of sustained reduction in ALT levels	Did not combine vitamin E with metformin to determine whether dual therapy could provide greater benefit	National Institute of Diabetes and Digestive and Kidney Diseases (NIDDK)
Vitamin E and hydroxytyrosol	NCT02842567 (67)	Vitamin E plus Hydroxytyrosol	Placebo	80 children with NAFLD aged 4–16	4 months	Improved steatosis, hypertriglyceridemia and systemic inflammation	Short follow-up duration	Bambino Gesù Hospital and Research Institute
Vitamin D	NCT02132442	Vitamin D (Ergocalciferol)	Placebo	12 children and adults with type 2 diabetes and NAFLD aged 10 to 50	6 months	Results not found	Small sample size, mixed age with adult	University of Massachusetts, Worcester
Combined treatment	NCT01934777 (61)	Omega-3 (DHA) plus Choline plus Vitamin E	Placebo	60 children with NAFLD aged 4–16	12 months	Improved steatosis and ALT and glucose levels	Small sample size, mixed age children and adolescent	Bambino Gesù Hospital and Research Institute
Combined treatment	NCT02098317 (62)	Omega-3 (DHA) plus Vitamin D	Placebo	66 children with NAFLD aged 4–16	12 months	Improved insulin resistance, lipid profile, ALT levels and NAS score	Small sample size, mixed age children and adolescent	Bambino Gesù Hospital and Research Institute

RCT, randomized controlled trial; MASLD, Metabolic Dysfunction-Associated Steatotic Liver Disease; PUFA, polyunsaturated fatty acid; DHA, Docosahexaenoic acid; NAFLD, Nonalcoholic fatty liver disease; AST, Aspartate transaminase; GGT, gamma-glutamyl transferase; APO-A1, Apolipoprotein A1; ALT, alanine aminotransferase; NAS, non-alcoholic Fatty Liver Disease Activity Score.

## 5 Discussion

The effective management of pediatric MASLD necessitates the involvement of a multidisciplinary team working in close synergy with the family. This team should include a Pediatric Hepatologist closely collaborating with a Nutrition Specialist as well as a Pediatric Psychologist. However, the cornerstone of this approach lies in its family-centered nature: the objective is not merely a question of “caring for the child” but of “caring for the family,” and this can only be achieved by fostering behavioral and lifestyle changes within the entire family unit.

Overall, the reported pediatric trials demonstrate a commendable effort in seeking additional treatments for MASLD, often using rigorous (placebo-controlled) study designs. However, the overall strength of the evidence is reduced by recurring limitations such as small sample sizes, short duration, and sometimes inconsistent results.

All studies focus on practical and accessible interventions such as diet and exercise, which represent the first line of treatment, combined with nutritional supplements. The studies we reported explore different dietary strategies, comparing different diets (low-sugar diet, Mediterranean diet, low-glycemic index diet, low-carbohydrate diet), which is crucial for understanding which strategy may be most effective. Some trials (e.g., NCT05073588, NCT04415112, NCT00480922) (25, 27) use an “active” control group (e.g., Mediterranean diet vs. hypocaloric or low-fat diet) rather than a simple usual diet group. This design is more robust because it allows for determining whether an intervention is superior to another established approach. These data, however, demonstrate that a low-sugar diet (NCT02513121) (20) and the Indo-Mediterranean diet (NCT05073588) play a role in improving steatosis and related metabolic parameters, and the addition of physical exercise (NCT02258126) (28) certainly confirms its contribution to improving steatosis, adiposity, and insulin resistance (26–28). In studies that evaluate not only behavioral strategies but also supplementation, the greatest strength is the use of a placebo-treated control group. Furthermore, some studies such as, study NCT00885313 (24 months) (59) and the TONIC study NCT00063635 (96 weeks) (80) provide valuable data on long-term effects, crucial for a chronic disease such as MASLD, while others show good results obtained from combined treatments (e.g., Omega-3 + Vitamin D or Omega-3 + Choline + Vitamin E) of multiple supplements which reflect a better potency in clinical practice, allowing to act on multiple mechanisms simultaneously (56, 60–62, 67, 78–80). In conclusion, non-pharmacological therapies currently represent the foundation of pediatric MASLD treatment. Targeted and personalized nutritional intervention, promotion of physical activity, weight management, and robust family and social psychological support, delivered within a multidisciplinary, family-focused framework, are essential to halting disease progression, improving medical outcomes, and promoting clinical well-being in MASLD children and adolescents.

Our analysis reaffirms the crucial importance of non-pharmacological therapies for pediatric MASLD. Although their benefits are evident, the quality of current scientific evidence offers room for improvement that must be urgently addressed. Future research priorities should focus on three key areas: implementing multicenter randomized clinical trials to ensure external validity of data, harmonizing evaluation criteria through the use of standardized outcome measures, and designing long-term follow-up studies to verify the stability of benefits. Strengthening these three areas is essential to translating the potential of these therapies into robust clinical recommendations. Although the journey may be long and arduous, early and sustained investment in these interventions offers hope not only for reversing liver damage but also for establishing lasting health outcomes into adulthood.
